# Catquest-9SF functioning over a decade – a study from the Swedish National Cataract Register

**DOI:** 10.1186/s40662-020-00220-4

**Published:** 2020-12-01

**Authors:** Mats Lundström, Maria Kugelberg, Per Montan, Ingela Nilsson, Madeleine Zetterberg, Konrad Pesudovs, Anders Behndig

**Affiliations:** 1 Department of Clinical Sciences/Ophthalmology, Faculty of Medicine, Lund University, Trossögatan 4, 37137 Karlskrona, Lund, Sweden; 2 Department of Clinical Neuroscience, Division of Ophthalmology and Vision, Karolinska Institutet, Stockholm, Sweden; 3 St Erik Eye Hospital, Stockholm, Sweden; 4 Capio Medocular, Lund, Sweden; 5 Department of Clinical Neuroscience, Institute of Neuroscience and Physiology, Sahlgrenska Academy at the University of Gothenburg, Gothenburg, Sweden; 6 University of New South Wales, Adelaide, SA Australia; 7 Department of Clinical Sciences/Ophthalmology, Umeå University, Umeå, Sweden

**Keywords:** Cataract extraction, Outcomes, Questionnaire, Patient-reported outcomes, Rasch analysis

## Abstract

**Background:**

The Swedish National Cataract Register (NCR) collects data on cataract surgery outcomes during March, including patient-reported outcomes using the Catquest-9SF questionnaire for over 11 years. Previous studies from NCR have shown that the preoperative visual acuity has improved over time. The main purpose of this study was to evaluate the Catquest-9SF Rasch scoring performance in this changing environment. A second purpose was to describe clinical data over the same period for those who completed the questionnaire.

**Methods:**

The performance of the Catquest-9SF was analysed by a separate Rasch analysis for each year, resulting in a preoperative and postoperative score for each participating patient in the annual cohorts. The clinical data and questionnaire scoring were analysed for each year in the period 2008–2018 inclusive.

**Results:**

Data were available for 42,023 eyes for 11 annual cohorts (2008–2018). The psychometric properties of the questionnaire were stable during the study period. Person separation (precision) for the whole period was 2.58 and varied between 2.45 and 2.72. The person reliability was 0.87 and varied between 0.86 and 0.88. The targeting of question difficulty to person ability became less accurate over time meaning that the item activities became easier to carry out without difficulty. The average targeting for the whole period was −2.06 and changed from −1.92 in 2008 to −2.31 in 2018. The person score improved both before surgery and after surgery, indicating that patients are undergoing surgery at a more able level and getting better outcomes. The average improvement by surgery decreased from 3.41 logits in 2008 to 3.21 logits in 2018 (*p* = 0.003).

Over time, patient age decreased from 75 to 74 years (*p* < 0.001) and the proportion of women decreased from 63.9 to 57.9% (*p* < 0.001). The mean preoperative visual acuity in both the operated eye and the better eye improved over time (0.47 to 0.40 logMAR, *p* < 0.001 and 0.22 to 0.19 logMAR, *p* < 0.001, respectively), as did the mean postoperative visual acuity in the operated eye (0.14 to 0.09 logMAR, *p* < 0.001).

**Conclusions:**

The Catquest-9SF retained stable psychometric properties over this 11-year period although more recent cohorts included slightly younger patients with somewhat better vision.

## Background

The Swedish National Cataract Register (NCR) [1] has used the Catquest-9SF questionnaire [2] regularly since 2008. The NCR collects data on outcomes of cataract surgery during March each year. Catquest-9SF is a Rasch scaled 9-item questionnaire measuring activity limitations in daily life due to poor vision because of cataract and is used by the NCR because of its documented responsiveness in cataract surgery [3, 4]. It has been translated to many languages and validated through Rasch analysis [5] and is recommended by the International Consortium of Health Outcomes Measurement [6] and the European Registry of Quality Outcomes for Cataract and Refractive Surgery [7].

As of 2018, the Catquest-9SF has remained unchanged since 2008.

It comprises 9 items (A, B, C1–C7) with 4 response options and the possibility to check “cannot decide” which is treated as missing data. Items A and C1–C7 are concerned with difficulty, and item B with satisfaction. Response options are coded 4 for “very great difficulty/very dissatisfied”, 3 for “great difficulty/fairly dissatisfied”, 2 for “some difficulty/fairly satisfied” and 1 for “no difficulty/very satisfied”. The clinical impression during these 11 years is that the indication for cataract surgery has slowly drifted towards better preoperative visual acuity. This change in preoperative visual acuity has been documented for three national registries where the Catquest-9SF is used: Sweden, the Netherlands and Malaysia [8]. Furthermore, the availability of cataract surgery in Sweden has improved over this period, with an increase in annual volume from about 75,000 procedures in 2008 to 133,000 in 2017 [9]. The question remains as to whether the psychometric characteristics of Catquest-9SF have been optimal for the changing clinical patient cohorts during this period.

The purpose of this study was to evaluate the Catquest-9SF Rasch scoring performance for cohorts with gradually increasing visual acuity and changing risk factors (ocular comorbidity, difficult surgery because of small pupil, white cataract, and previous eye surgery) [8] over 11 years. We think this is of general interest, given the widespread use of the questionnaire, and also of specific interest to cataract surgeons who may want to adopt the questionnaire.

## Methods

Surgeons and clinics are invited by the NCR to participate in March every year on a voluntary basis. About 80% of all clinics reporting preoperative data to NCR also take part in the outcome data collection by reporting data on consecutive surgeries for March. Preoperative data include age, sex, visual acuity of both eyes, previous cataract surgery in the fellow eye, and ocular co-morbidities (age-related macular degeneration, glaucoma, diabetic retinopathy, and “other” sight-threatening eye disease) in the operated eye. Visual acuity values in original decimal notation were transformed to logMAR values for the statistical analysis. Surgical data include laterality (eye), perioperative difficulties (small pupil with need of stretching, white or dense cataract with need of staining, need of capsular hooks and capsular tension ring), type of surgery, type of intra-ocular lens (IOL) and surgical complication (posterior capsule rupture). Outcome data include preoperative K values, axial length, IOL calculation formula, visual acuity, planned refraction and refractive outcome (refraction).

**Fig. 1:**
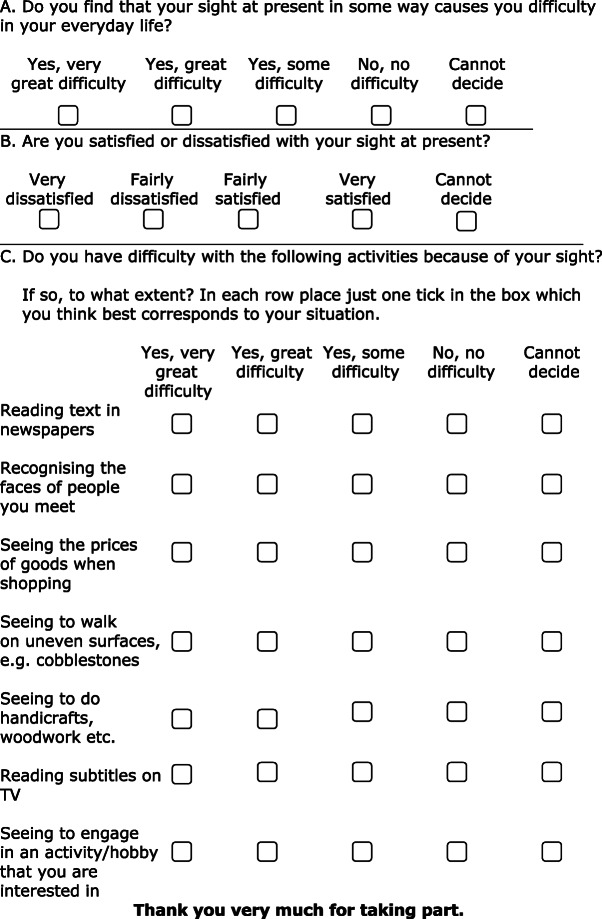
The English version of the Catquest-9SF form

Most of the clinics reporting outcome data during March also use the Catquest-9SF questionnaire (Fig. 1) [2]. Patients are asked to fill in the Catquest-9SF on a voluntary basis before cataract surgery and 3 months after surgery. The preoperative questionnaire is completed on paper at the clinic after surgery has been decided on, either during the preoperative examination or on the day of surgery. Patients who are unable to understand the questionnaire or do not understand Swedish are excluded from this part of the outcome study. The postoperative questionnaire is completed at home as a postal paper form with a prepaid envelope or on the web using unique per-patient login credentials. More than 90% of patients operated during March in participating clinics completed the preoperative questionnaire, and between 70 and 80% of these patients returned the postoperative questionnaire.

### Rasch analysis

Precision is a crucial characteristic of all measurement tools. In Rasch analysis, precision is calculated as person separation and person reliability. These entities should have values above 2.0 and 0.80, respectively, to show good precision. This means an ability to separate the respondents in at least three strata of visual function*.* These precision values provide an evaluation of how the questionnaire performs along the continuum of raw score measure for the whole test. The person separation reflects the number of levels of ability that the questionnaire can separate the respondents into, while the person reliability is a measure of the questionnaire’s internal consistency. Targeting is a measure of how well the difficulty of the items matches the ability of the patients to endorse the items.

In a Rasch analysis, each item is given a Rasch score for the response options. A mean Rasch score for each item can then be calculated based on the scoring of the response options, corresponding to the difficulty of the item for the study cohort. A negative Rasch item score signifies a more difficult item to endorse, while a positive value indicates the opposite. This is related to the raw score polarity (with 4 meaning very great difficulty and 1 meaning no difficulty). In Rasch analysis, item scoring and person scoring are calculated and analysed on the same single linear scale that uses a logit (natural logarithm of the odds ratio) unit.

### Statistical analysis

Rasch analysis was performed using version 4.2.0 of Winsteps (Chicago, IL, USA). A Rasch analysis was performed independently for each annual data cohort. Statistical analyses of clinical data and Rasch person scores were performed using version 25.0 of IBM SPSS Statistics (IBM Corp., Armonk, NY, USA). Testing included bivariate Pearson correlation, t-tests, and linear regression. Trends over time were analysed with a Chi-squared test for categorical data and ANOVA for continuous data. A *p*-value of < 0.05 was considered significant.

The study adhered to the tenets of the Declaration of Helsinki. The Swedish Data Inspection Board approved the data collection in the NCR and an ethics vetting board approved the collection of information in the Catquest-9SF questionnaire.

## Results

The number of follow-up cases versus number of completed preoperative and postoperative Catquest-9SF is shown in Table 1.

**Table 1:** Number of cases with follow-up data and completed Catquest-9SF during March 2008 to 2018 inclusive

Surgery year	Number of patients with follow-up data	Number (%) of patients with completed Catquest-9SF before and after surgery
2008	1296	845 (65.2)
2009	6960	3023 (43.4)
2010	6549	3815 (58.3)
2011	6463	3514 (54.4)
2012	6118	3716 (60.7)
2013	6512	3666 (56.3)
2014	7954	4601 (57.8)
2015	6796	4484 (66.0)
2016	7031	4472 (63.6)
2017	8636	5312 (61.5)
2018	6908	4575 (66.2)
Total	71,223	42,023 (59.0)

### Patients

Patient characteristics in terms of age, sex, preoperative visual acuity, first- or second-eye surgery and postoperative visual acuity in the annual cohorts are shown in Table 2. The mean follow-up time in each year varied between 31 and 41 days with an overall mean of 36 days.

**Table 2:** Number of patients, preoperative characteristics, and postoperative visual acuity

Year	Number of patients	Female (%)	Mean age (years)	2nd eye surgery (%)	Mean preoperative BCVA operated eye (logMAR)	Mean preoperative BCVA better eye (logMAR)	Ocular co-morbidity^a^ (%)	Mean post-operative BCVA, operated eye (logMAR)
2008	845	63.9	75.5 ± 9.1	41.3	0.47	0.22	32.3	0.14
2009	3023	60.3	75.8 ± 9.1	43.0	0.48	0.23	32.8	0.12
2010	3815	61.4	74.5 ± 9.0	40.2	0.49	0.22	35.6	0.12
2011	3514	61.8	74.5 ± 9.1	40.6	0.45	0.21	34.5	0.11
2012	3716	60.6	74.3 ± 8.7	40.0	0.45	0.24	39.0	0.12
2013	3666	60.1	74.0 ± 8.7	41.7	0.43	0.22	39.0	0.10
2014	4601	59.9	74.1 ± 8.6	38.7	0.44	0.20	39.2	0.11
2015	4484	59.4	74.2 ± 8.2	37.1	0.41	0.23	39.5	0.10
2016	4472	59.6	74.2 ± 8.1	36.4	0.41	0.19	36.0	0.10
2017	5312	59.1	74.9 ± 8.1	35.3	0.42	0.19	39.9	0.10
2018	4575	57.9	74.7 ± 7.9	37.7	0.40	0.19	37.4	0.09
P (trend)		*P* < 0.001^b^	*P* < 0.001^c^	*P* < 0.001^b^	*P* < 0.001^c^	*P* < 0.001^c^	*P* < 0.001^b^	*P* < 0.001^c^

*BCVA* = best corrected visual acuity; *logMAR* = logarithm of the minimum angle of resolution

^a^Ocular comorbidity includes age-related macular degeneration, glaucoma, diabetic retinopathy, and “other” sight- threatening ocular comorbidity

^b^Chi-squared test

^c^One-way ANOVA

Preoperative visual acuity and age in groups are outlined in Table 3.

**Table 3:** Visual acuity in two groups and age in two groups per year

Year	Preoperative visual acuity, logMAR ≥0.0 (≥20/20) N (%)	Preoperative visual acuity, logMAR≥0.3 (≥20/40) N (%)	Age ≤ 65 years N (%)	Age ≥ 66 years N (%)	Missing data N (%)	Total number of patients
2008	10 (1.2)	368 (43.6)	131 (15.5)	714 (84.5)	0	845
2009	24 (0.8)	1252 (41.4)	427 (14.1)	2582 (85.4)	14 (0.5)	3023
2010	28 (0.7)	1517 (39.8)	607 (15.9)	3208 (84.1)	0	3815
2011	24 (0.7)	1658 (47.2)	532 (15.1)	2969 (84.5)	13 (0.4)	3514
2012	52 (1.4)	1775 (47.8)	529 (14.2)	3145 (84.6)	42 (1.1)	3716
2013	55 (1.5)	1853 (50.5)	558 (15.2)	3073 (83.8)	35 (1.0)	3666
2014	62 (1.3)	2261 (49.1)	641 (13.9)	3959 (86.0)	1 (0)	4601
2015	50 (1.1)	2241 (50.0)	570 (12.7)	3879 (86.5)	35 (0.8)	4484
2016	72 (1.6)	2438 (54.5)	558 (12.5)	3875 (86.7)	39 (0.9)	4472
2017	77 (1.4)	2995 (56.4)	561 (10.6)	4750 (89.4)	1 (0)	5312
2018	64 (1.4)	2682 (58.6)	493 (10.8)	4021 (87.9)	61 (1.3)	4575

### Description of data

The annual precision values together with the number of completed questionnaires and targeting are shown in Table 4.

**Table 4:** Number of completed questionnaires, questionnaire precision (person separation and person reliability) and targeting

Year	N (completed questionnaires)	Person separation	Person reliability	Targeting: person mean score (item = 0)
2008	1690	2.57	0.87	−1.92
2009	6046	2.67	0.88	−1.87
2010	7630	2.68	0.88	−1.81
2011	7028	2.72	0.88	−1.69
2012	7432	2.61	0.87	−1.87
2013	7332	2.54	0.87	−2.07
2014	9202	2.58	0.87	−2.08
2015	8968	2.57	0.87	−2.10
2016	8944	2.54	0.87	−2.19
2017	10,624	2.55	0.87	−2.16
2018	9150	2.45	0.86	−2.31

**Fig. 2:**
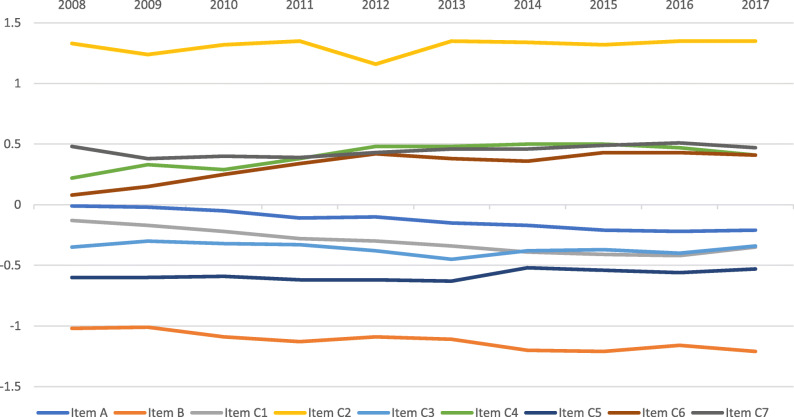
Average item Rasch score per item, 2008 to 2018 inclusive. The item Rasch score corresponds to the difficulty of the item. A more negative value signifies a more difficult item to endorse “no difficulty” for

For the whole study period, the person separation was 2.58 and the person reliability was 0.87. There was a tendency of misfit for item C4 (Seeing to walk on uneven ground) and item C2 (Recognizing the faces of people you meet). For item C4 and item C2, fit statistics showed Infit mean square–Outfit mean square of 1.27–1. 42 and 1.38–1.33, respectively. For the other 7 items, Infit mean square varied between 0.70 and 1.08 while Outfit mean square varied between 0.72 and 1.03. The average targeting for the whole study period was −2.06, the preoperative targeting was −0.45 logits and the postoperative targeting was −3.61 logits. Differential item functioning (DIF) was tested for sex and surgery year. There was no DIF over 0.5 logits for any item versus these two parameters. A mean score for each item is shown for all study years in Fig. 2.

**Fig. 3:**
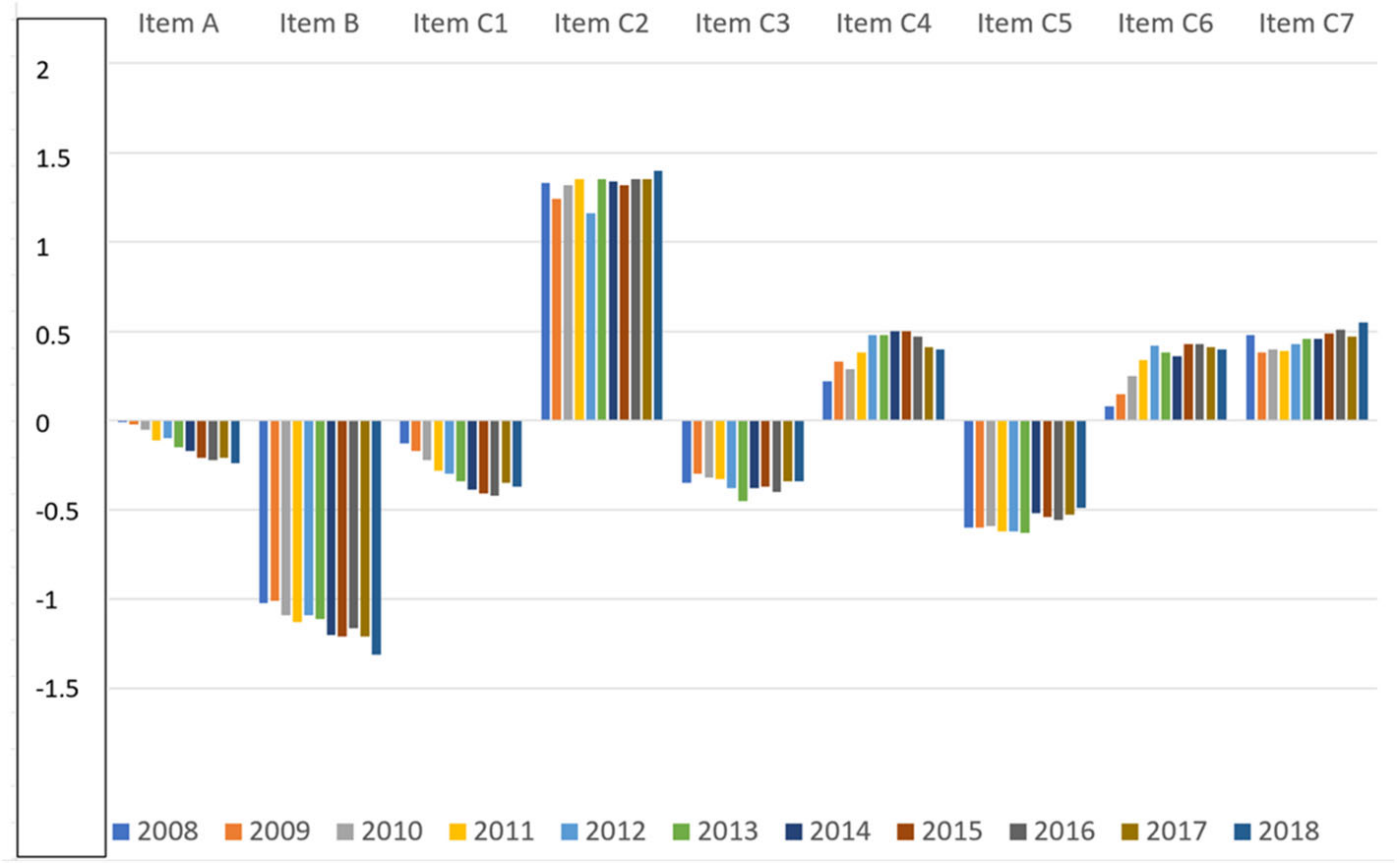
Item average scoring per year, 2008 to 2018 inclusive. The figure shows how the level of difficulty for each item has performed during the study period

The pattern of the average item Rasch scoring over time is shown in Fig. 3, revealing how the level of difficulty for each item varied during the study period.

**Fig. 4:**
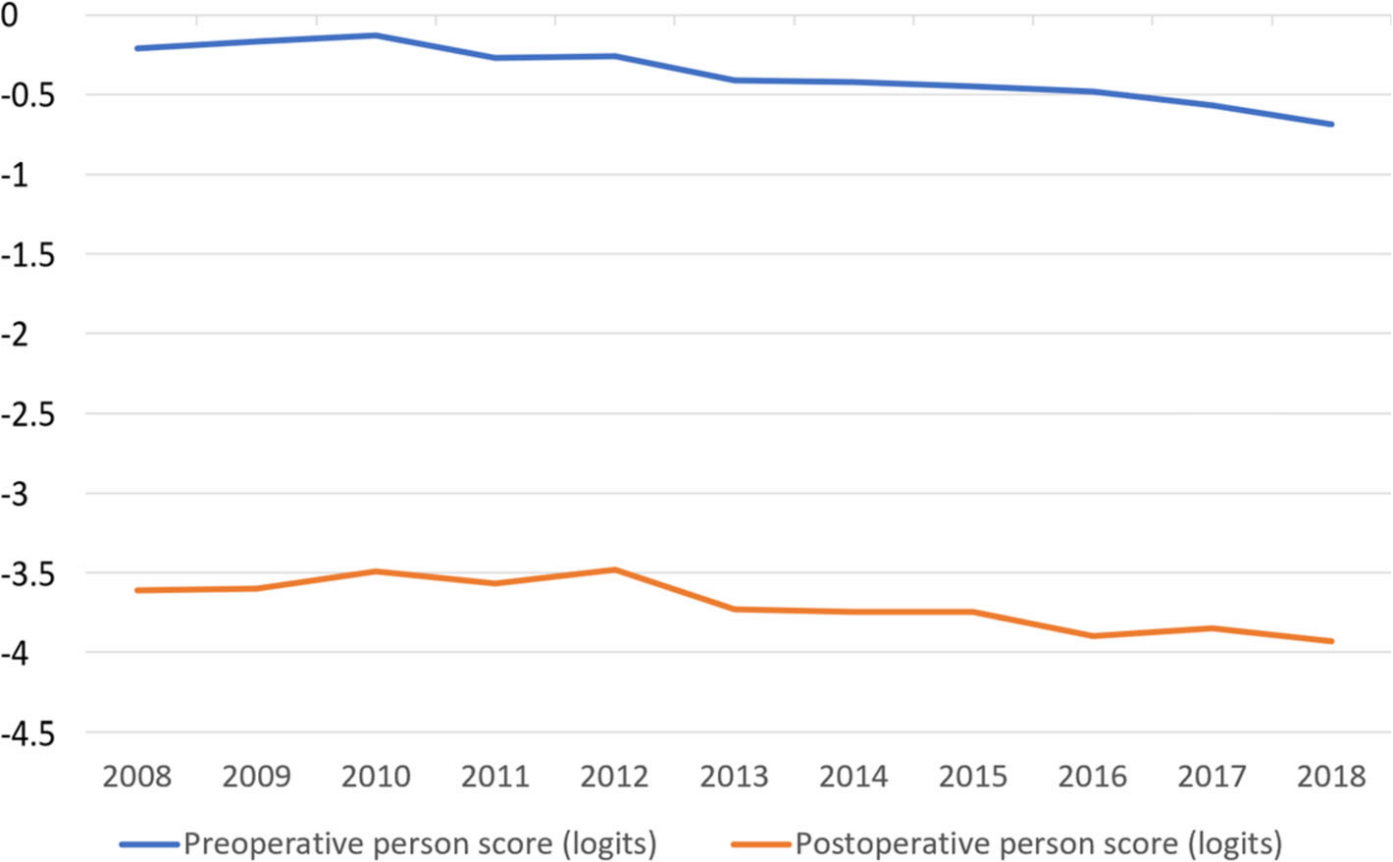
Average person Rasch score before surgery (blue) and after surgery (yellow) over time. A more negative value means less activity limitation

Figures 2 and 3 show that items A, B and C1 change over time towards more negative Rasch item scoring which means that they are easier to endorse for the patients. The mean preoperative and postoperative Rasch person score over time is shown in Fig. 4. The Rasch person score varied between − 6.45 and 5.92 in this study. A more negative value signifies less difficulty in carrying out the requested item activities in daily life. The average improvement (difference between preoperative and postoperative Rasch person score) by surgery decreased from 3.41 logits in 2008 to 3.21 logits in 2018 (*p* = 0.003).

### Analyses

The bivariate Pearson correlation coefficient between preoperative Rasch person score and visual acuity (logMAR) in the operated eye, was −0.213 (*p* < 0.01) for the whole period, varying between −0.165 and −0.222 (*p* < 0.01) for each year. The bivariate Pearson correlation coefficient between preoperative Rasch person score and visual acuity (logMAR) better eye, was −0.283 (*p* < 0.01) for the whole period. For each year, it varied between −0.184 and −0.309 (*p* < 0.01).

Single variable analyses of preoperative Rasch person score showed a significantly higher mean score (i.e., more perceived difficulty) for women compared to men (−0.22 vs. −0.68, *p* < 0.001, t-test), for patients with an ocular comorbidity compared to those with no ocular comorbidity (−0.19 vs. −0.53, *p* < 0.001, t-test), for first-eye surgery compared to second-eye surgery (−0.20 vs. −0.72, *p* < 0.001, t-test) and for patients with surgical difficulty compared to those with no surgical difficulty (−0.06 vs. −0.43, *p* < 0.001, t-test). The differences were statistically significant for every year in all analyses, except for 2008 in the sex comparison and for 3 out of the 11 years in the surgical difficulty analysis. The bivariate Pearson correlation coefficient between preoperative Rasch person score and age was −0.046 (*p* < 0.01) for the whole period. When age was dichotomized into < 75 and ≥ 75, the younger age group had a significantly higher preoperative Rasch person score (−0.30 vs. −0.50, *p* < 0.001, t-test). This difference existed for analysis of each single study year but was not always statistically significant (data not shown). Table 5 shows a linear stepwise regression analysis with preoperative person Rasch score as dependent variable and all other parameters included as independent variables. All independent variables were significantly related to the preoperative person Rasch score.

**Table 5:** Linear stepwise regression analysis of all data, 2008 to 2018 inclusive

Variable	Unstandardized beta	Standard Error	Standardized beta	Significance	95% CI, lower	95% CI, upper
BCVA in the better eye	3.399	0.053	0.320	0.000	3.294	3.503
Age	−0.033	0.001	−0.138	0.000	−0.036	−0.031
Sex	−0.406	0.019	−0.096	0.000	−0.444	−0.368
Surgery year	−0.047	0.003	−0.066	0.000	−0.053	−0.040
Ocular comorbidity^a^	0.211	0.020	0.049	0.000	0.172	0.251
Surgical difficulty^b^	0.216	0.037	0.027	0.000	0.143	0.289
Second-eye surgery	−0.109	0.021	−0.026	0.000	−0.149	−0.068

Dependent variable: preoperative person Rasch score. Independent variables: preoperative BCVA (logMAR) in the better eye, age, sex (coded as female 0, male 1), surgery year, ocular co-morbidity (coded as yes 1, no 0), surgical difficulty (coded as yes 1, no 0) and second-eye surgery (coded as yes 1, no 0)

*BCVA* = best corrected visual acuity; *logMAR* = logarithm of the minimum angle of resolution; *95% CI* = 95% confidence interval

^a^Ocular comorbidity includes age-related macular degeneration, glaucoma, diabetic retinopathy, and “other” sight-threatening ocular comorbidity

^b^Surgical difficulty includes small pupil with need of stretching, white or dense cataract with need of staining, need of capsular hooks and capsular tension ring

In Table 5, unstandardized beta shows the size of change of the dependent variable for every 1-unit change of the independent variable. To interpret this correctly, it is important to remember that improvement of the dependent variable means a more negative value and that a negative beta means a decrease and a positive beta indicates an increase of the dependent variable.

The same analysis was also conducted for each single year cohort separately. Preoperative visual acuity in the better eye and age were both significantly related to the preoperative person Rasch score in all single study years. Ocular co-morbidity, second-eye surgery, and sex were also significantly related to the preoperative person Rasch score in all years except for ocular comorbidity in 2009 and second-eye surgery and sex in 2008. Surgical difficulty was only significantly related to the preoperative person Rasch score in 2016.

The bivariate Pearson correlation coefficient between postoperative Rasch person score and postoperative visual acuity (logMAR) in the operated eye, was −0.355 (*p* < 0.001) for the whole period and varied between −0.292 and −0.417 (*p* < 0.001) for each year. Table 6 shows a linear stepwise regression analysis with postoperative person Rasch score as dependent variable and preoperative characteristics and capsule complication included as independent variables. Capsule complication included posterior capsular break with or without vitreous loss. All independent variables were significantly related to the postoperative person Rasch score.

**Table 6:** Linear stepwise regression analysis of all data, 2008–2018 inclusive

Variable	Unstandardized beta	Standard error	Standardized beta	Significance	95% CI, lower	95% CI, upper
BCVA in the operated eye	4.138	0.063	0.331	0.000	4.014	4.262
Ocular comorbidity^a^	0.414	0.024	0.087	0.000	0.367	0.461
Second-eye surgery	−0.362	0.023	−0.076	0.000	−0.406	−0.317
Sex	−0.178	0.023	−0.038	0.000	−0.222	−0.134
Capsule complication	0.483	0.122	0.019	0.000	0.244	0.722
Age	−0.004	0.001	−0.013	0.007	−0.006	−0.001
Surgical difficulty^b^	−0.112	0.044	−0.012	0.011	−0.198	−0.026

Dependent variable: postoperative person Rasch score. Independent variables: postoperative best corrected visual acuity (logMAR) in the operated eye, age, sex (coded as female 0, male 1), ocular co-morbidity (coded as yes 1, no 0), surgical difficulty (coded as yes 1, no 0), second-eye surgery (coded as yes 1, no 0), and capsule complication (coded as yes 1, no 0)

*BCVA* = best corrected visual acuity; *logMAR* = logarithm of the minimum angle of resolution; *95% CI* = 95% confidence interval

^a^Ocular comorbidity includes age-related macular degeneration, glaucoma, diabetic retinopathy, and “other” sight threatening ocular comorbidity

^b^Surgical difficulty includes small pupil with need of stretching, white or dense cataract with need of staining, need of capsular hooks and capsular tension ring

The same analysis was also conducted for each single year cohort separately. Postoperative visual acuity in the operated eye was significantly related to the postoperative person Rasch score in all single study years. Ocular co-morbidity and second-eye surgery were also significantly related to postoperative person Rasch score in all years except 2008. The other independent variables were significantly related to postoperative person Rasch score occasionally for single years.

## Discussion

The relationship between demographic data, risk factors (co-existing eye diseases and surgical difficulty), first- or second-eye surgery, and Rasch scoring will reflect how the performance of the Catquest-9SF is influenced by changing patient cohorts. The annual cohorts showed changes in preoperative visual acuity and risk factors (Table 2). Mean age and proportion of women reported in the study both decreased over the years, in line with previous results [1]. A better preoperative visual acuity over time for patients undergoing cataract surgery has been documented in other cohorts [9]. Our study cohorts showed a decreasing frequency of second-eye surgery (*p* < 0.001, univariate analysis of variance) and an increasing frequency of ocular co-morbidity (*p* < 0.001, univariate analysis of variance). The other risk factors in terms of surgical difficulties (data not shown) did not show any conclusive trend.

The precision of the Catquest-9SF remained acceptable over time (Table 4). The person separation coefficient should be over 2.0 for an acceptable precision [2, 4, 10], and this limit was surpassed every year with a reasonable margin. Targeting, on the other hand, showed an increasing discrepancy between item mean difficulty (set to zero) and the mean person ability. The mean person ability changed from −1.92 to −2.31 over time, indicating an increasing ability for the patients to endorse the items. This is partly due to postoperative questionnaires being included in the analysis as a successful cataract extraction resolves all activity limitations caused by cataract, but probably also indicates an increasing visual acuity over time after surgery (Table 1). Figures 2 and 3 show the background for an increasingly poor targeting over time; item A, item B and item C1 show a statistically significant (*p* < 0.001) change in Rasch scoring, suggesting that these items are increasingly easier for the respondents to endorse. One way to counteract this development could be to include more difficult items in a future revision of the questionnaire. One area that has been suggested is the performance of several activities involving electronic devices which have become routine in recent years [11]. Many activity limitation cataract surgery questionnaires show low levels of difficulties pre-surgery, reflecting the fact that many of these questions were designed two to three decades ago when the surgery was performed at later stages of the disease as compared with present times. Rasch analysis provides insight into question targeting and has been used to re-validate most cataract surgery questionnaires [12]. Another way to counteract the poor targeting is to use item-banking [13, 14]. Other Rasch analysis outputs such as ordered response options and, dimensionality are not reported in the present article, but for each year they were within acceptable limits, as has been described in previous studies among different combinations of these cohorts [2, 15–18].

Some item scores converged to clusters over time (Fig. 2). The easiest item was item C2 (recognizing faces). Easy items were item C4 (walking on uneven ground), item C6 (reading subtitles on TV), and item C7 (doing hobby activities). More difficult items were item C1 (reading newspapers), item A (having problems in general), item C3 (seeing prices), and item C5 (doing handicrafts). The most difficult item was item B (satisfaction with vision). This means that items for near vision formed one cluster and items for distance vision formed another cluster over time.

The difficulty (item score) of the 9 items showed a similar pattern over the 11 study years (Fig. 3).

The average activity limitations for subjects (person Rasch score) in the annual cohorts gradually diminished over time (Fig. 4) both before surgery (blue line) and after surgery (yellow line). We believe that one reason for this change over time was better visual acuity (Tables 2 and 3).

To test the criterion validity of the Catquest-9SF, a comparison with other similar questionnaires would be desirable. However, no other questionnaires were used for these cohorts during the study period. One alternative is to make a comparison with clinical data; specifically, testing the correlation between Catquest-9SF scoring and visual acuity could be used as a proxy for similar questionnaires. Catquest-9SF measures activity limitations in daily life because of poor vision and so visual acuity is the most natural parameter for comparison with the person Rasch score for determining criterion validity. The Pearson correlation coefficient showed a significant correlation between preoperative visual acuity of the eye to be operated on and the preoperative person Rasch score. This correlation was even stronger between preoperative visual acuity of the better eye and preoperative Rasch score. This finding corresponds well with what was found in the first Catquest-9SF test in Sweden [19].

As shown in Table 5, the regression analysis indicated that preoperative visual acuity in the better eye was the most influential parameter on preoperative person Rasch score. However, other patient characteristics were also related to the preoperative person Rasch score in the total study cohort (Table 5) and for some single-year cohorts. Lower age, existence of an ocular co-morbidity, female sex, first-eye surgery, and existence of eye characteristics that may cause surgical difficulty were all related to higher Rasch person score (i.e., a less negative numeric value), indicating more perceived problems in daily life compared with the opposite situation.

The postoperative person Rasch score was significantly related to postoperative visual acuity of the operated eye (Table 6), and for single years it was also related to ocular co-morbidity and second-eye surgery. Age, sex, and surgical difficulties were less closely related to the postoperative person Rasch score. The relationship between ocular co-morbidity and poor patient-reported outcome of activity limitations has also been described for another questionnaire [20]. In recent years, cataract surgery has offered an opportunity to treat refractive errors by use of non-monofocal intraocular lenses. This makes another dimension interesting for patient-reported outcomes, namely visual symptoms [21].

A weakness of our study is the selection of clinics and patients based on one calendar month and on a voluntary participation**.** Another weakness is that we do not know what information are hidden among the non-responders, which may affect the generalizability of our findings. A strength is the large number of completed questionnaires over a decade.

## Conclusion

The Catquest-9SF had stable and reliable psychometric properties over 11 years of use, although the annual patient cohorts showed slightly improving mean visual acuity and slightly decreasing mean age. Future improvement could be achieved by introducing new items to replace groups of old items with similar properties. On the other hand, reducing the number of items too far may influence the precision, which is an important quality for a questionnaire [22].

## Data Availability

The datasets generated and analysed during the current study are not publicly available due to integrity issues but are available from the corresponding author on reasonable request.
